# Spleen tyrosine kinase Syk is critical for sustained leukocyte adhesion during inflammation in vivo

**DOI:** 10.1186/1471-2172-8-31

**Published:** 2007-11-28

**Authors:** David Frommhold, Ingrid Mannigel, Jürgen Schymeinsky, Attila Mocsai, Johannes Poeschl, Barbara Walzog, Markus Sperandio

**Affiliations:** 1 Children's Hospital, Neonatal Unit, University of Heidelberg, Germany; 2 Walter Brendel Center of Experimental Medicine, Ludwig-Maximilians-University München, Germany; 3 Department of Physiology, Semmelweis University School of Medicine, Budapest, Hungary

## Abstract

**Background:**

During inflammation, β_2_-integrins mediate leukocyte adhesion to the endothelium accompanied by the activation of the spleen tyrosine kinase Syk.

**Results:**

We investigated leukocyte adhesion and rolling in cremaster muscle venules before and during stimulation with fMLP using mice with a *Syk*^-/- ^hematopoietic system. In unstimulated venules, *Syk*^-/- ^leukocytes adhered less efficiently than control leukocytes while rolling was similar between *Syk*^-/- ^and control leukocytes. During fMLP-superfusion, control mice showed significantly increased adhesion accompanied by reduced rolling. For *Syk*^-/- ^leukocytes, an increase in adhesion with a concomitant decrease in rolling was only observed during the first three minutes during fMLP stimulation, but not at later time points. We also investigated leukocyte spreading against the vessel wall during fMLP stimulation and found a significant impairment of spreading for *Syk*^-/- ^leukocytes. Additional in vitro experiments revealed that the adhesion and spreading defect seen in *Syk*^-/- ^chimeric mice was due to compromised β_2_-integrin-mediated outside-in signaling.

**Conclusion:**

We provide substantial evidence for an important role of Syk in mediating β_2_-integrin dependent outside-in signaling leading to sustained leukocyte adhesion and spreading during the inflammatory response in vivo.

## Background

The recruitment of leukocytes into inflamed tissue is an important immunological process, which proceeds along a well-defined cascade of events beginning with the capture of leukocytes to the inflamed endothelium and followed by leukocyte rolling along the endothelium [1]. During rolling, leukocytes are in intimate contact with the inflamed endothelium enabling endothelial bound chemokines to interact with specific chemokine receptors expressed on the leukocyte surface. This in turn triggers the activation of β_2_-integrins with subsequent leukocyte arrest [2]. According to the current paradigm of integrin-dependent leukocyte adhesion, pro-inflammatory factors such as chemokines or the formyl-peptide fMLP mediate intracellular signaling events, preferentially via G-protein coupled receptors, which induce an increase in affinity and avidity of β_2_-integrins towards their ligands (inside-out signaling) [2,3]. The concomitant engagement of activated β_2_-integrins through their ligands results in lateral clustering of integrins leading to various intracellular responses that regulate rearrangement of the cytoskeleton, migratory behavior, and survival (outside-in signaling) [4,5]. Previous studies have demonstrated a crucial role of the non-receptor spleen tyrosine kinase Syk for β_2_-integrin dependent signaling in neutrophils [6-8]. Mocsai et al. used wild type mice with a *Syk*^-/- ^hematopoietic system and found that stimulation of neutrophils with fMLP led to a normal response concerning respiratory burst, degranulation of primary and secondary granules, and activation of ERK and p38 MAPK [7]. Similar results have been found after stimulation with chemokines macrophage-inflammatory-protein-2 and -1α (MIP-2, MIP-1α), leukotriene LTB4 and the complement factor C5a [7]. These responses were dependent on G-protein coupled receptors and analysed under conditions where integrin-dependent signaling was low or absent (most of the experiments were conducted without Mg^2+^). Hence, it was concluded that Syk is not required for signaling events mediated by G-protein coupled receptors. On the other hand, a recent report from Zarbock and colleagues who used an ex vivo flow chamber system, uncovered a novel neutrophil activation pathway which is independent of G-protein-coupled receptors but requires P-selectin glycoprotein ligand-1 (PSGL-1) dependent signaling mediated via Syk which leads to partial activation of the β_2_-integrin LFA-1 (inside-out signaling) resulting in a significant slowing down in leukocyte rolling velocity without influencing firm leukocyte arrest [9]. Concerning the involvement of Syk in outside in signaling events, Mocsai et al. reported defective integrin-dependent functions in *Syk*^-/- ^chimeric mice [6]. In that study, neutrophils were exposed to immobilized fibrinogen, recombinant ICAM-1 or the integrin-binding RGD-motif of human fibronectin upon stimulation by tumor necrosis factor-α (TNF-α). This led to a substantial production of superoxide anions in wild type but not in CD18^-/- ^neutrophils. *Syk*^-/- ^neutrophils also failed to manifest increased superoxide anion production upon TNF-α-stimulation when plated on various integrin ligand surfaces [6]. In addition, the same authors demonstrated that adhesion-dependent degranulation and spreading of murine neutrophils were severely reduced in *Syk*^-/- ^neutrophils upon stimulation by TNF-α [6]. Interestingly, additional in vitro and in vivo neutrophil migration assays did not reveal any defects in neutrophil migration. In the thioglycollate-induced peritonitis model, a well established in vivo assay to investigate leukocyte recruitment, the extravasation of *Syk*^-/- ^neutrophils was as efficient as that of control cells tested within the same animal (containing a mixed chimeric hematopoietic system of both *Syk*^-/- ^and control cells) [6]. These findings are in contrast with a recent report from Schymeinsky et al., who demonstrated that emigration of neutrophils in fMLP-stimulated cremaster muscle tissue is significantly impaired in mice with a *Syk*^-/- ^hematopoietic system [10] suggesting that leukocyte emigration is significantly influenced by the stimulus used and/or the type of tissue studied.

To elucidate the in vivo relevance of Syk-mediated signaling on leukocyte recruitment and potentially distinguish between the contributions of inside-out signaling and outside-in signaling on leukocyte recruitment in respect to Syk, we set out to investigate leukocyte rolling and adhesion in unstimulated and fMLP-stimulated cremaster muscle venules in mice with a *Syk*^-/- ^hematopoietic system. To unequivocally allocate an outside-in signaling defect in the absence of Syk, we also studied CD18-mediated adhesion in a static in vitro assay using isolated *Syk*^-/- ^neutrophils plated onto immobilized fibrinogen in the presence of Mn^2+^, which is known to induce a high-affinity state of β_2_-integrins [11]. The results from our study present substantial evidence that during inflammation in vivo the non-receptor tyrosine kinase Syk is crucial for firm and sustained leukocyte adhesion mediated via integrin-dependent outside-in signaling.

## Results

### Leukocyte adhesion and rolling in unstimulated cremaster muscle venules

We investigated leukocyte rolling and adhesion in unstimulated and fMLP-stimulated cremaster muscle venules of four mice containing a *Syk*^-/- ^hematopoietic system and six *Syk*^+/- ^mice (referred to as control mice) using intravital microscopy. Microvascular parameters are presented in Table 1 and showed no significant difference in vessel diameter, blood flow velocity, and wall shear rate between *Syk*^-/- ^chimeric mice and control mice. However, we observed significantly higher systemic leukocyte counts in *Syk*^-/- ^chimeric mice compared to control mice (Table 1).

**Table 1:** Microvascular parameters (mean ± SEM; diameter, centerline velocity, shear rate, and WBC) of cremaster muscle venules of control mice and Syk^-/- ^chimeric mice.

	**Mice**	**Venules**	**Diameter**	**Centerline Velocity**	**Shear Rate**	**WBC**
	[n]	[n]	[μm]	[μm/s]	[1/s]	[/μl]

***Syk***^-/- ^***chimeric***	4	13	32 ± 1.3	1,800 ± 150	1,400 ± 100	5,700 ± 450
***Control***	6	26	34 ± 1.0	2,100 ± 200	1,500 ± 100	3,200 ± 250
			n.s. ^a)^	n.s.	n.s	p < 0.05^b)^

^a) ^n.s., not significant; ^b) ^indicates significant differences at p < 0.05.

**Figure 1:**
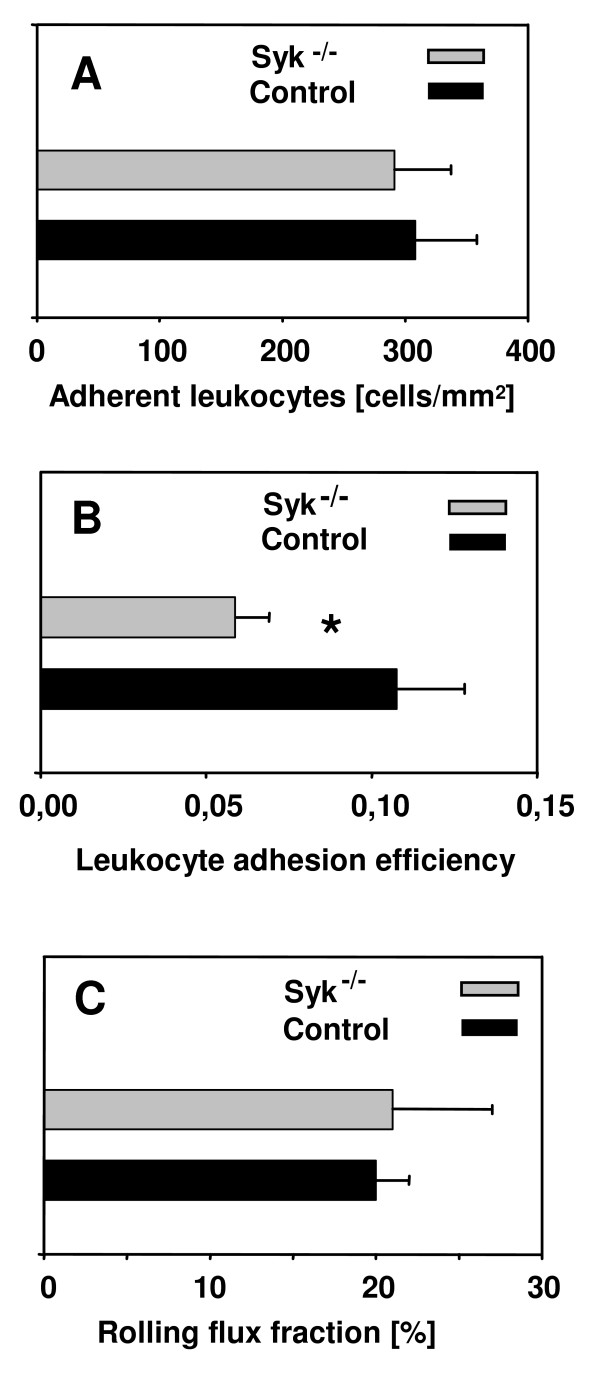
**Leukocyte adhesion and rolling (mean ± SEM) in unstimulated cremaster muscle venules**. Number of adherent leukocytes [adherent cells/mm^2 ^vessel surface area] (A), leukocyte adhesion efficiency [(adherent cells/mm^2 ^vessel surface area)/(systemic leukocyte count)] (B), and rolling flux fraction [%] (C) in *Syk*^-/- ^chimeric mice (gray bar, n = 4) and control mice (black bar, n = 6). * indicates significant difference (p < 0.05) between *Syk*^-/- ^chimeric and control mice.

In the present study, leukocyte adhesion in unstimulated cremaster muscle venules was similar between *Syk*^-/- ^chimeric mice (290 ± 50 cells/mm^2^) and control mice (310 ± 50 cells/mm^2^) observed within the first 30 min after exteriorization of the cremaster muscle (Fig. 1A). As leukocyte adhesion is greatly influenced by the availability of leukocytes within the vasculature, we also calculated the efficiency of leukocyte adhesion by dividing the number of adherent cells by the number of circulating leukocytes. We found that adhesion efficiency of *Syk*^-/- ^leukocytes (0.06 ± 0.01) was significantly decreased when compared to control leukocytes (0.11 ± 0.02) (Figure 1B), suggesting that Syk is involved in mediating firm and sustained leukocyte adhesion to inflamed endothelium in vivo.

Next, we analyzed leukocyte rolling in unstimulated cremaster muscle venules of *Syk*^-/- ^chimeric mice within the first 30 min after exteriorization of the cremaster muscle. Leukocyte rolling in this setting is induced by surgical trauma and completely dependent on P-selectin [12]. Similar to Zarbock and colleagues [9], but in contrast to in vitro work by Abbal et al. [13], we found no difference in leukocyte rolling between *Syk*^-/- ^chimeric mice (rolling flux fraction of 21% ± 6%) and control mice (rolling flux fraction 20% ± 2%) (Fig. 1C) suggesting no obvious functional defect in P-selectin dependent rolling in this setting.

### Leukocyte adhesion and rolling following local stimulation with fMLP

As described above, we have shown that leukocyte adhesion efficiency is decreased in *Syk*^-/- ^chimeric mice in vivo (Figure 1B). Several in vitro studies using human neutrophils have suggested that Syk is involved in signaling events following engagement of β_2_-integrins by their ligands (outside-in signaling). In addition, its role in the preceding intracellular signals leading to the activation of β_2_-integrins (inside-out signaling) has been demonstrated not to influence firm leukocyte adhesion [9]. From this we hypothesized that during local stimulation with the pro-inflammatory mediator fMLP, leukocyte adhesion caused by inside-out signaling (leading to activated β_2_-integrins) should be similar between *Syk*^-/- ^chimeric mice and control mice. In contrast, during sustained leukocyte arrest, which is dependent on integrin mediated outside-in signaling, leukocyte adhesion should be reduced in *Syk*^-/- ^chimeric mice when compared to control mice.

**Figure 2:**
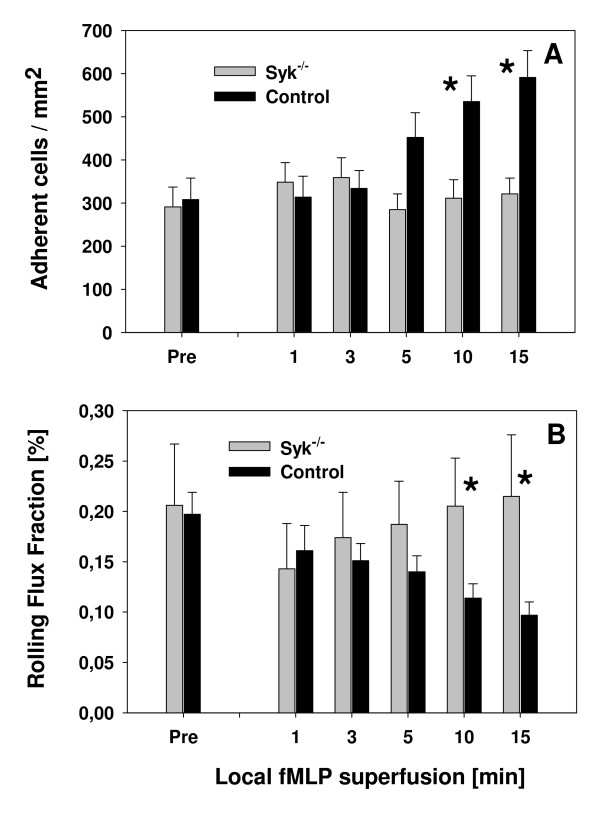
**Leukocyte adhesion and rolling (mean ± SEM) during local stimulation of cremaster muscle venules with fMLP**. Leukocyte adhesion [number of adherent cells/mm^2^] (A) and leukocyte rolling flux fraction [%] (B) in *Syk*^-/- ^chimeric mice (gray bar, n = 4)) and control mice (black bar, n = 6) before and during 15 min local administration of fMLP (1 μM). * indicates significant difference (p < 0.05) between *Syk*^-/- ^chimeric and control mice.

To test the above-formulated hypothesis, we locally stimulated the cremaster muscle microcirculation with the formyl-peptide fMLP (1 μM). As depicted in Figure 2A and 2B, we found a similar increase in the number of adherent leukocytes during the first three minutes of fMLP superfusion accompanied by an initial decrease in leukocyte rolling suggesting that fMLP-induced activation of β_2_-integrins leading to increased binding to their ligands does not require Syk. Interestingly, at later time points (≥ 5 min), a substantial increase in leukocyte adhesion with a marked decrease in leukocyte rolling was only observed in control mice while the number of adherent and rolling *Syk*^-/- ^leukocytes returned to baseline levels (Fig. 2A and 2B). These results clearly suggest that for sustained firm leukocyte arrest, Syk-dependent mechanisms are required.

### Postarrest deformation of adherent leukocytes

**Figure 3:**
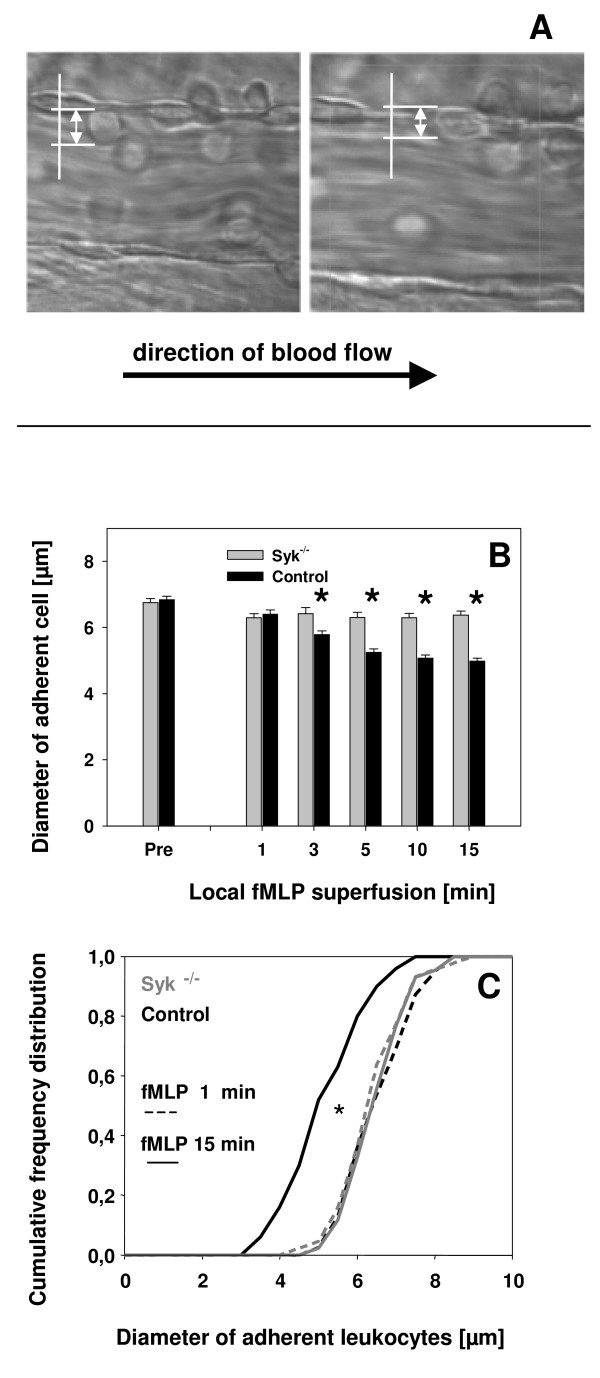
**Cell diameter changes of adherent leukocytes (mean ± SEM) during local stimulation of cremaster muscle venules with fMLP**. Microphotographs of an adherent control leukocyte immediately after attachment (A, left panel) and during gradual stimulation (A, right panel) leading to flattening out of the cell with a concomitant decrease in cell diameter perpendicular to the vessel wall (white arrows). Diameters of adherent leukocytes from *Syk*^-/- ^chimeric mice (gray bar, n = 318 from 4 mice) and control mice (black bar, n = 419 from 6 mice) were measured perpendicular to the vessel wall before and during superfusion with fMLP (B). In addition, a cumulative frequency distribution of measured leukocyte diameters is given for *Syk*^-/- ^(gray lines) and control leukocytes (black lines) after 1 min (dashed lines) and 15 min (solid lines) fMLP superfusion (C). * in (B): significant difference (p < 0.05) between *Syk*^-/- ^chimeric and control mice. * in (C): significant difference (p < 0.05) in the distribution of control cell diameters at 15 min fMLP to all other groups.

Hirahashi and colleagues have recently identified a crucial role of the β_2_-integrin Mac-1 and Syk in an in vivo model of thrombohemorrhagic vasculopathy induced by a local Shwartzman reaction [14]. In that study, they observed by histology a more pronounced flattening (spreading) of neutrophils to the inflamed vessel wall in control mice compared to Mac-1 deficient mice. As Syk is required for the Mac-1 dependent vasculopathy [14], we analysed shape changes (spreading) of adherent leukocytes in fMLP-stimulated cremaster muscle venules by measuring cell diameter perpendicular to the vessel wall during superfusion with fMLP. To distinguish an immediate leukocyte arrest from the Mac-1 dependent postarrest step, we measured adherent leukocyte diameters perpendicular to the vessel surface. Immediately after attachment, leukocytes from control and *Syk*^-/- ^bone marrow chimeric mice presented mostly as round shaped cells (3A, left panel). During gradual activation, leukocytes from control mice deformed and spread out along the vascular wall (3A, right panel) while this was significantly impaired in *Syk*^-/- ^bone marrow chimeric mice. Diameters of adherent *Syk*^-/- ^leukocytes (6.8 μm ± 0.1 μm) assessed before fMLP were not significantly different from diameters of adherent control leukocytes (6.7 μm ± 0.2 μm). During fMLP stimulation, mean cell diameter of attached control cells gradually decreased – an indication of spreading out against the vessel wall – while diameters of attached *Syk*^-/- ^leukocytes did not significantly change (Figure 3B). Comparing cell diameters between *Syk*^-/- ^and control leukocytes at 1, 3, 5, 10, and 15 min fMLP stimulation revealed that a significant difference in cell diameter between the groups became evident at ≥ 3 min fMLP treatment (Figure 3B). These results suggest that spreading of leukocytes against the vessel wall is Syk-dependent and requires signals, which are distinct from those leading to the initial attachment of leukocytes to the vessel wall. This is also illustrated in the cumulative frequency distribution of cell diameters assessed at 1 min and 15 min fMLP treatment. At 1 min fMLP, we did not find a significant difference in the frequency distribution of cell diameters between *Syk*^-/- ^and control leukocytes (Figure 3C). However, after 15 min treatment with fMLP, attached control leukocytes had significantly flattened out while adherent *Syk*^-/- ^leukocytes still had a similar cell diameter distribution as observed in adherent *Syk*^-/- ^and control leukocytes at 1 min fMLP (Figure 3C). These results indicate that spreading of attached leukocytes is markedly impaired in the absence of Syk and therefore confirm an important role of Syk in integrin-mediated outside-in signaling preparing the attached cell for the successful transmigration through the vessel wall.

### Leukocyte adhesion and spreading in vitro

**Figure 4:**
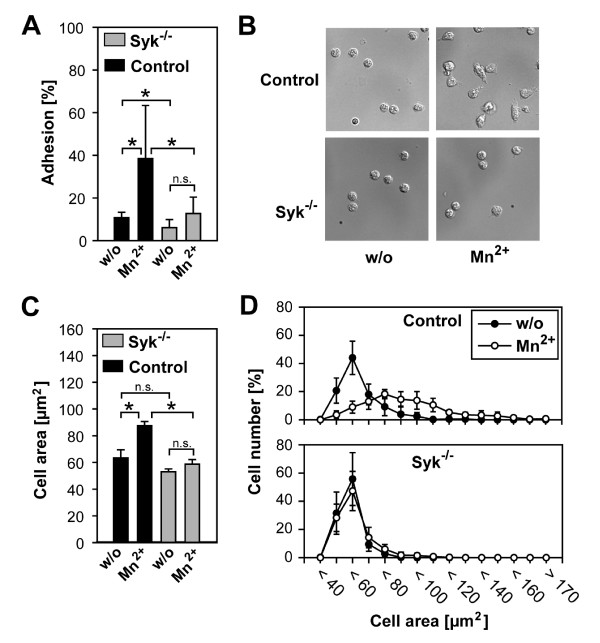
**Neutrophil adhesion and spreading (mean ± SD) on immobilized fibrinogen**. Adhesion and spreading of isolated *Syk*^-/- ^(gray bar) or control neutrophils (black bar) with or without (w/o) addition of 1 mM Mn^2+ ^at 37°C for 30 min. Adherent *Syk*^-/- ^(n = 7 mice) or control neutrophils (n = 5 mice) in percent of total cells added (A), microscopic images (B), increase of cell area (in μm^2^, C) and frequency distribution of cell area (D) of adherent *Syk*^-/- ^(n = 400 from 4 mice) and control neutrophils (n = 400 from 4 mice) upon stimulation for 30 min at 37°C. * indicates significant difference (p < 0.05), n.s., not significant.

To further investigate the crucial role of Syk for sustained leukocyte adhesion and spreading, we performed additional static in vitro studies on isolated neutrophils obtained from mice with a *Syk*^-/- ^hematopoietic system or control animals in the presence of Mn^2+ ^upon exposure to immobilized fibrinogen, a native ligand of the β_2_-integrins Mac-1 and gp150/95. Because the treatment of PMN with Mn^2+ ^favors the high-affinity state of β_2_-integrins in the absence of inside-out signaling [11], this reductionist approach enabled us to specifically investigate the role of Syk for outside-in signaling (Figure 4A). We found that Mn^2+ ^significantly increased adhesion of unstimulated control neutrophils from 10.8 ± 2.5% to 38.8 ± 24.9% (p < 0.05) of total cells added, whereas *Syk*^-/- ^neutrophils only showed a slight but not statistically significant increase in adhesiveness (6.1 ± 3.8% versus 12.8 ± 7.6%, n.s.). Of note, under unstimulated conditions (without Mn^2+^), the number of adherent *Syk*^-/- ^neutrophils was significantly lower than the number of adherent control neutrophils (p < 0.05), which is in line with our in vivo findings (Figure 1B).

Next, we analyzed neutrophil spreading. Control neutrophils underwent substantial spreading within 30 min after addition of Mn^2+ ^leading to an increase of cell area from 60 ± 5 μm^2 ^without Mn^2+ ^to 89 ± 3 μm^2 ^with Mn^2+ ^(Figure 4B and 4C). In contrast, spreading of *Syk*^-/- ^neutrophils did not significantly increase (53 ± 2 μm^2 ^without Mn^2+ ^vs. 56 ± 3 μm^2 ^with Mn^2+^). Moreover, analysis of cell spreading by calculating the frequency distribution of the cell area did not only reveal a diminished capability but a complete inability of the majority of *Syk*^-/- ^neutrophils to spread out onto immobilized fibrinogen, which demonstrates that Syk is indispensable for β_2_-integrin mediated cell function (Figure 4D).

## Discussion

Many studies have indicated a role of Syk in neutrophil activation during an inflammatory response [6-8]. While most of the studies suggested a participation of Syk in integrin-dependent neutrophil activation, a recent report also provided substantial evidence that Syk is involved in PSGL-1 dependent partial activation of LFA-1 (inside-out signaling), which leads to significant changes in leukocyte rolling velocity without affecting firm leukocyte adhesion [9]. We have investigated the in vivo relevance of Syk-mediated signaling on leukocyte recruitment in vivo by observing leukocyte rolling and adhesion in unstimulated and fMLP-stimulated cremaster muscle venules in mice with a *Syk*^-/- ^hematopoietic system. We found that leukocyte adhesion efficiency in unstimulated cremaster muscles of *Syk*^-/- ^chimeric mice was significantly reduced under baseline conditions compared to wild type mice although the absolute number of adherent cells was similar in both groups. The apparent difference between adhesion and adhesion efficiency can be explained by the significant increase in systemic leukocyte count in Syk-deficient bone marrow chimeras. This increase could be due to a latent infection caused by the low B-cell count in *Syk*^-/- ^bone marrow chimeric mice. However, it is unlikely, that the low B-cell count contributed to the impaired adhesion and spreading since any subclinical infection/inflammation caused by the short-term deficiency of B-cells would facilitate, rather than inhibit neutrophil adhesion and spreading.

To further elucidate the contribution of Syk in leukocyte adhesion, we performed local superfusion of the cremaster muscle with fMLP, a pro-inflammatory agent, which acts on neutrophils through a specific G-protein coupled pathway. Interestingly, we found an increase in the number of adherent leukocytes in *Syk*^-/- ^chimeric mice early during fMLP superfusion, which was similar to that observed in control mice. These in vivo results expand those reported earlier by our group [10], in as much as they indicate that Syk is not required for the initial arrest of leukocytes to the fMLP-stimulated endothelial lining, which is triggered by inside-out signaling events. Similar results were reported by Zarbock et al. using a microflow chamber system [9]. Their study did not find a difference in firm leukocyte arrest after six minutes of perfusion of whole blood from control mice vs. mice pretreated with the Syk-inhibitor piceatannol through the micro-flow chamber coated with E-selectin and ICAM-1 [9]. However, in contrast to the unaffected leukocyte adhesion at early time points during stimulation with fMLP, we demonstrated that later during fMLP superfusion, a further significant increase in leukocyte adhesion was absent in *Syk*^-/- ^chimeric mice but present in control mice. These results indicate a role of Syk in the recently suggested Mac-1-dependent postarrest step during leukocyte recruitment, which is required to prepare the attached cell for its extravasation into tissue and depends on outside-in signaling events [14]. Because leukocyte spreading on the inflamed endothelium may be considered being part of the proposed postarrest step, we investigated leukocyte spreading during fMLP stimulation and found a significant impairment in spreading in the absence of Syk. To our knowledge, this is the first direct in vivo observation of a Syk-dependent spreading defect. Similarly, additional static in vitro experiments using control and *Syk*^-/- ^neutrophils treated with Mn^2+ ^(leading to a shift in β_2_-integrin activation towards a high affinity state) demonstrated that leukocyte adhesion and spreading were significantly impaired in the absence of Syk, which confirms the important role of Syk in mediating outside-in signaling dependent processes during leukocyte recruitment.

We also have assessed leukocyte rolling in unstimulated and fMLP-stimulated cremaster muscle venules. Similar to the in vivo findings by Zarbock et al. [9], but in contrast to the report from Abbal et al. [13], we did not see an impairment in P-selectin dependent rolling. Using a flow chamber assay and the acute myelogenous human leukemia cell line KG1, Abbal and co-workers found that rolling of KG1 cells on immobilized P-selectin was strongly impaired, if KG1 cells were pretreated with the Syk inhibitor piceatannol or with Syk-specific siRNA [13]. In case of piceatannol, there is substantial evidence that piceatannol is a rather unspecific inhibitor of Syk, as it also acts on Src-family kinases and members of the focal adhesion kinase (FAK) family [15,16]. This raises the possibility that Syk-independent effects of piceatannol may interfere with P-selectin mediated rolling. Haller et al. demonstrated that P-selectin binding to T-lymphocytes leads to tyrosine phosphorylation of pp125 focal adhesion kinase (FAK) [17]. FAK is a substrate for Src and has been reported to phosphorylate paxillin, an important adaptor protein which is known to associate with α_4_-integrin to establish firm cell adhesion under shear flow conditions [18]. However, Syk has also been reported to associate with P-selectin glycoprotein ligand-1 (PSGL-1), the main if not only relevant P-selectin ligand in vivo [19]. The association is mediated via the actin-linking ERM (ezrin/radixin/moesin) proteins enabling Syk-dependent signaling following engagement of PSGL-1 [19]. Although we cannot rule out any influence of Syk on P-selectin mediated rolling as observed by Abbal et al. in their in vitro assays, our in vivo results and those from Zarbock et al. demonstrate that the abrogation of Syk does not lead to a relevant reduction in P-selectin dependent rolling in vivo [9].

## Conclusion

Our in vivo experiments provide substantial evidence for a relevant role of Syk in firm and sustained β_2_-integrin-mediated leukocyte adhesion as well as spreading of leukocytes against the vessel wall during inflammation in vivo. In addition, we demonstrate that rapid activation of β_2_-integrins leading to the initial binding to integrin ligands on the inflamed endothelium in vivo does not require Syk. Finally, these results confirm the recently suggested expansion of the leukocyte adhesion cascade [20,21], distinguishing an initial leukocyte arrest step (triggered by inside-out signaling) from a postarrest, adhesion-strengthening step, which requires outside-in signaling via β_2_-integrins. Therefore, Syk does not only influence leukocyte recruitment during leukocyte rolling, an early event in the adhesion cascade, but also substantially influences the preparation of already attached leukocytes in finding their way out of the microvasculature into inflamed tissue.

## Methods

### Animals and generation of bone marrow chimeric mice

*Syk*^+/- ^mice carrying the S*yk*^tm1Tyb ^mutation were obtained from Victor Tybulewicz (National Institute of Medical Research, London, UK) [22] and kept on the C57BL/6 genetic background (which carries the CD45.2 allele). Bone marrow chimeras with a *Syk*^-/- ^or *Syk*^+/- ^(referred to as control group) hematopoietic system were generated by fetal liver transplantation as described [6]. Briefly, *Syk*^-/- ^and control fetal liver cell suspensions were prepared from E15.5-E17.5 fetal livers obtained from timed mating of *Syk*^+/- ^carriers. Recipient mice carrying the CD45.1 allele on the C57BL/6 genetic background (Jackson Laboratory, Bar Harbor, ME, USA) were lethally irradiated by 11 Gy from a ^60^Co source and injected intravenously with Syk^-/- ^or control fetal liver cell suspensions. 4–6 weeks after transplantation, the repopulation of the hematopoietic system by donor-derived cells was confirmed by flow cytometric analysis of CD45.2 expression in peripheral blood neutrophils (Gr1-positive gate) and/or the absence of B220-positive cells in the *Syk*^-/- ^chimeras [22]. These studies indicated that the repopulation of the hematopoietic system by donor-derived cells was consistently more than 95%. Bone marrow chimeras were used for experiments 6–8 weeks after transplantation. Mice were kept in individually ventilated cages in a conventional facility. All animal experiments were approved by the Regierungspräsidium Karlsruhe, Germany, AZ 35-9185.81/G-67/03 or the Semmelweis University Animal Experimentation Review Board, Budapest, Hungary, 883/003/2005.

### Intravital microscopy

Mice were anesthetized with intraperitoneal (i.p.) injection of ketamine (125 mg/kg body weight, Ketanest, Pfizer, Karlsruhe, Germany) and xylazine (12.5 mg/kg body weight; Phoenix Scientific, Inc., St. Joseph, MO) and placed onto a heating pad to maintain body temperature at 37°C. Intravital microscopy was conducted on an upright microscope (Leitz, Wetzlar, Germany) with a saline immersion objective (SW 40/0.75 numerical aperture). To ease breathing, mice were intubated using PE 90 tubing (ID: 0.86 mm, OD: 1.27 mm; Becton Dickinson, Heidelberg, Germany). The left carotid artery was cannulated with PE 10 tubing (ID: 0.28 mm, OD: 0.61 mm, Becton Dickinson) for blood sampling. During the experiment mice received 0.2 ml/h normal saline i.a. to maintain neutral fluid balance. The surgical preparation of the cremaster muscle for intravital microscopy was performed as previously described [23]. Briefly, after opening the scrotum, the cremaster muscle was mobilized and spread over a cover glass. The epididymis and testis were moved to the side giving full microscopic access to the cremaster muscle microcirculation. Experiments were recorded via a CCD camera system (model CF8/1; Kappa, Gleichen, Germany) on a Panasonic S-VHS recorder. The cremaster muscle was superfused with a thermocontrolled (35°C) bicarbonate-buffered saline (131.9 mM NaCl, 18 mM NaHCO_3_, 4.7 mM KCl, 2.0 mM CaCl_2_·2H_2_O, and 1.2 mM MgCl_2_) equilibrated with 5% CO_2 _in N_2_. For local treatment with N-formyl-Met-Leu-Phe (fMLP; Sigma, Deisenhofen, Germany), 1 μM fMLP was added to the superfusion buffer and administered onto the preparation over 15 min. Postcapillary venules under observation ranged from 20–40 μm in diameter and were recorded before and during fMLP administration. Systemic blood samples (10 μl) were taken and assessed for white blood cell count before and after the experiment. Blood samples were diluted 1:10 with Türck's solution (Merck, Darmstadt, Germany) and leukocyte concentration was expressed as number of leukocytes per microliter of whole blood using a hematocytometer.

### Data analysis of intravital experiments

Vessel diameter, leukocyte diameter, and vessel segment length of postcapillary venules were measured using a digital image processing system [24]. To assess spreading of adherent leukocytes against the vessel wall, diameter of attached leukocytes was measured perpendicular to the vessel wall before and at various time points during fMLP superfusion. As illustrated in Figure 5, a decrease in perpendicular diameter indicates spreading out against the vessel wall. Centerline red blood cell velocities in postcapillary venules of the cremaster muscle were assessed by a dual photodiode and a digital on-line cross-correlation program (Circusoft Instrumentation, Hockessin, USA) and converted to mean blood flow velocities as described [25]. Wall shear rates (γ_w_) were estimated as reported previously [26,27]. Rolling leukocyte flux fraction was defined as the percentage of rolling leukocytes to all leukocytes passing the same vessel in one minute [25]. The number of adherent leukocytes was assessed as adherent cells per mm^2 ^vessel surface area, and leukocyte adhesion efficiency defined as number of adherent leukocytes per mm^2 ^vessel surface area/systemic leukocyte count [28].

### In vitro adhesion and spreading assay

Murine bone marrow neutrophils were isolated from femurs and tibias as reported earlier and suspended (10^5^/sample) in adhesion medium (HEPES buffer supplemented with 0.25% BSA, 0.1% glucose, 1.2 mM Ca^2+ ^and 1 mM Mg^2+^) [10]. Next, cells were plated onto fibrinogen (250 μg/ml)-coated 96-well microtiter plates (Greiner, Frickenhausen, Germany). After stimulation of the cells by 1 mM Mn^2+ ^for 30 min at 37°C, non-attached cells were rinsed away and attached cells were stained using 0.1% Crystal Violet (Sigma, Deisenhofen, Germany) after fixation with 1% glutaraldehyde [29] and measured in triplicates using a microplate reader (Tecan, Crailsheim, Germany). Spreading on fibrinogen-coated cover slips (Saur, Reutlingen, Germany) was analyzed after fixation of the neutrophils by 3.7% formaldehyde using a Zeiss 200 M microscope with a Plan-Apochromat 63×/1.4 oil objective (Zeiss, Göttingen, Germany) and an AxioCam HR digital camera (Zeiss, Göttingen, Germany). Photoshop 7 software was used to create panels of the recorded images (Adobe, San Jose, CA, USA). Analysis of cell spreading was carried out off-line in four independent experiments by measuring 100 cells from each experiment using ImageJ version 1.33 provided by the National Institutes of Health, USA .

### Statistics

Sigma Stat 2.0 software package (SPSS Science, Chicago, IL) was used for statistical analysis. Vessel diameters, leukocyte diameter, blood flow velocities, shear rates, leukocyte counts, rolling flux fraction, the number of adherent leukocytes, and leukocyte adhesion efficiency in wild type and *Syk*^-/- ^chimeric mice were compared with the one-way ANOVA on ranks (Kruskal-Wallis) with a multiple pairwise comparison test (Dunn's test) or Student's t-test, as appropriate. Statistical significance was set at *p *< 0.05, indicated by *.

## Abbreviations

Syk, spleen tyrosine kinase.

## Authors' contributions

The authors contributed to the work as following: DF and IM collected data, analyzed data, and performed research; JS performed research and contributed to the preparation of the manuscript; JP contributed to the preparation of the manuscript; AM contributed analytical tools (Syk-deficient bone marrow chimeras); BW designed research and contributed to the preparation of the manuscript, and MS designed research, analyzed data, and wrote the paper.

## References

[B1] Springer TA (1995). Traffic signals on endothelium for lymphocyte recirculation and leukocyte emigration. Annu Rev Physiol.

[B2] Laudanna C, Alon R (2006). Right on the spot. Chemokine triggering of integrin-mediated arrest of rolling leukocytes. Thromb Haemost.

[B3] Luo BH, Carman CV, Springer TA (2007). Structural Basis of Integrin Regulation and Signaling. Annu Rev Immunol.

[B4] Kim M, Carman CV, Yang W, Salas A, Springer TA (2004). The primacy of affinity over clustering in regulation of adhesiveness of the integrin alphaL beta2. J Cell Biol.

[B5] Whitlock BB, Gardai S, Fadok V, Bratton D, Henson PM (2000). Differential roles for alpha(M)beta(2) integrin clustering or activation in the control of apoptosis via regulation of akt and ERK survival mechanisms. J Cell Biol.

[B6] Mocsai A, Zhou M, Meng F, Tybulewicz VL, Lowell CA (2002). Syk is required for integrin signaling in neutrophils. Immunity.

[B7] Mocsai A, Zhang H, Jakus Z, Kitaura J, Kawakami T, Lowell CA (2003). G-protein-coupled receptor signaling in Syk-deficient neutrophils and mast cells. Blood.

[B8] Berton G, Mocsai A, Lowell CA (2005). Src and Syk kinases: key regulators of phagocytic cell activation. Trends Immunol.

[B9] Zarbock A, Lowell CA, Ley K (2007). Spleen Tyrosine Kinase Syk Is Necessary for E-Selectin-Induced alpha(L)beta(2) Integrin-Mediated Rolling on Intercellular Adhesion Molecule-1. Immunity.

[B10] Schymeinsky J, Sindrilaru A, Frommhold D, Sperandio M, Gerstl R, Then C, Mocsai A, Scharffetter-Kochanek K, Walzog B (2006). The Vav binding site of the non-receptor tyrosine kinase Syk at Tyr 348 is critical for beta2integrin (CD11/CD18)-mediated neutrophil migration. Blood.

[B11] Diamond MS, Springer TA (1994). The dynamic regulation of integrin adhesiveness. Curr Biol.

[B12] Ley K, Bullard DC, Arbones ML, Bosse R, Vestweber D, Tedder TF, Beaudet AL (1995). Sequential contribution of L- and P-selectin to leukocyte rolling in vivo. J Exp Med.

[B13] Abbal C, Lambelet M, Bertaggia D, Gerbex C, Martinez M, Arcaro A, Schapira M, Spertini O (2006). Lipid raft adhesion receptors and Syk regulate selectin-dependent rolling under flow conditions. Blood.

[B14] Hirahashi J, Mekala D, Van Ziffle J, Xiao L, Saffaripour S, Wagner DD, Shapiro SD, Lowell C, Mayadas TN (2006). Mac-1 signaling via Src-family and Syk kinases results in elastase-dependent thrombohemorrhagic vasculopathy. Immunity.

[B15] Mocsai A, Jakus Z, Vantus T, Berton G, Lowell CA, Ligeti E (2000). Kinase pathways in chemoattractant-induced degranulation of neutrophils: the role of p38 mitogen-activated protein kinase activated by Src family kinases. J Immunol.

[B16] Law DA, DeGuzman FR, Heiser P, Ministri-Madrid K, Killeen N, Phillips DR (1999). Integrin cytoplasmic tyrosine motif is required for outside-in alphaIIbbeta3 signalling and platelet function. Nature.

[B17] Haller H, Kunzendorf U, Sacherer K, Lindschau C, Walz G, Distler A, Luft FC (1997). T cell adhesion to P-selectin induces tyrosine phosphorylation of pp125 focal adhesion kinase and other substrates. J Immunol.

[B18] Alon R, Feigelson SW, Manevich E, Rose DM, Schmitz J, Overby DR, Winter E, Grabovsky V, Shinder V, Matthews BD, Sokolovsky-Eisenberg M, Ingber DE, Benoit M, Ginsberg MH (2005). Alpha4beta1-dependent adhesion strengthening under mechanical strain is regulated by paxillin association with the alpha4-cytoplasmic domain. J Cell Biol.

[B19] Urzainqui A, Serrador JM, Viedma F, Yanez-Mo M, Rodriguez A, Corbi AL, Alonso-Lebrero JL, Luque A, Deckert M, Vazquez J, Sanchez-Madrid F (2002). ITAM-based interaction of ERM proteins with Syk mediates signaling by the leukocyte adhesion receptor PSGL-1. Immunity.

[B20] Zhang H, Schaff UY, Green CE, Chen H, Sarantos MR, Hu Y, Wara D, Simon SI, Lowell CA (2006). Impaired integrin-dependent function in Wiskott-Aldrich syndrome protein-deficient murine and human neutrophils. Immunity.

[B21] Ley K, Zarbock A (2006). Hold on to your endothelium: postarrest steps of the leukocyte adhesion cascade. Immunity.

[B22] Turner M, Mee PJ, Costello PS, Williams O, Price AA, Duddy LP, Furlong MT, Geahlen RL, Tybulewicz VL (1995). Perinatal lethality and blocked B-cell development in mice lacking the tyrosine kinase Syk. Nature.

[B23] Sperandio M, Thatte A, Foy D, Ellies LG, Marth JD, Ley K (2001). Severe impairment of leukocyte rolling in venules of core 2 glucosaminyltransferase-deficient mice. Blood.

[B24] Klyscz T, Junger M, Jung F, Zeintl H (1997). Cap image--a new kind of computer-assisted video image analysis system for dynamic capillary microscopy. Biomed Tech (Berl).

[B25] Sperandio M, Pickard J, Unnikrishnan S, Acton ST, Ley K (2006). Analysis of leukocyte rolling in vivo and in vitro. Methods Enzymol.

[B26] Long DS, Smith ML, Pries AR, Ley K, Damiano ER (2004). Microviscometry reveals reduced blood viscosity and altered shear rate and shear stress profiles in microvessels after hemodilution. Proc Natl Acad Sci U S A.

[B27] Smith ML, Long DS, Damiano ER, Ley K (2003). Near-wall micro-PIV reveals a hydrodynamically relevant endothelial surface layer in venules in vivo. Biophys J.

[B28] Dunne JL, Ballantyne CM, Beaudet AL, Ley K (2002). Control of leukocyte rolling velocity in TNF-alpha-induced inflammation by LFA-1 and Mac-1. Blood.

[B29] Kueng W, Silber E, Eppenberger U (1989). Quantification of cells cultured on 96-well plates. Anal Biochem.

